# Molecular Mass and Isoelectric Point Analysis of Cytokinin Sequences in the Wheat Genome

**DOI:** 10.3390/ijms26115270

**Published:** 2025-05-30

**Authors:** Meshal M. Almutairi, Saad M. Alzahrani

**Affiliations:** Advanced Agricultural and Food Technology Institute, King Abdulaziz City for Science and Technology (KACST), P.O. Box 6086, Riyadh 11442, Saudi Arabia; smzahrni@kacst.gov.sa

**Keywords:** wheat, chromosome sequences, amino acids, molecular weight (grams/mole), theoretical isoelectric point, relative codon bias strength (RCBS), principal component analysis (PCA)

## Abstract

Cytokinins play an important role in plants and are targets of wheat breeding, particularly in terms of flowering and yield. The objective of this study was to determine relative synonymous codon usage (RSCU), molecular weight (g/mol), theoretical isoelectric point, instability index, aliphatic index, and hydrophobicity for the wheat cytokinin sequences from two different databases. The methods employed involved different formulas for calculations. The relative synonymous codon usage values were calculated as the ratio of the observed frequency to the expected frequency for the particular codon. The theoretical isoelectric point was calculated based on dissociation constant for groups of carboxylic acid and amino acids groups. The results showed that values of the relative synonymous codon usage divided amino acids of wheat into two groups. In the first group, values were above 1.6 (significant overrepresentation), such as those for phenylalanine (TTC), and Leucine (TTA). In the second group, values were below 0.6 (underrepresentation) such as those for leucine (CTA) and valine (GTT). In addition, the theoretical isoelectric point (pI) ranged from 4.81 to 6.6, and the instability index values were 34.3 and 38.16. A high degree of instability was observed at 1D and 5D of wheat genomes with values of 54.16 and 50.36, respectively. Principal component analysis (PCA) of the RSCU revealed that the main variation was attributed to PC1, accounting for a total variation of about 72.11%. The amino acids contributing to this variation included isoleucine, leucine, lysine, aspartic acid, and serine. PCA of the theoretical isoelectric point results found that the main variation was attributed to PC1, with a total variation of about 58.88%, and these chromosomes included 5D, 4D, 1A, 4B, and 3D of wheat genomes. Understanding the importance of RSCU in plant breeding helps breeders understand the mechanisms and functional aspects of wheat genomes, thereby enabling the development of wheat genomes for environmental adaptations. These results will provide a reference for nutrition and industrial applications, as well as supporting breeding programs.

## 1. Introduction

As one of most important plant hormone groups, cytokinins play significant roles and are targets of wheat breeding, particularly in terms of flowering and yield [1,2], as well as mitigating environmental stress (including biotic and abiotic stresses) [3]. The metabolic cycling of cytokinin involves initial steps catalyzed by isopentenyl transferase enzymes to form cytokinins, thereby activating them into activating free-base forms by lonely guy enzymes (LOG) [4]. The pathway of signaling cytokinins involves multi-step phosphorylation systems that comprise histidine kinases and histidine phosphotransfer proteins [5]. In wheat, cytokinins cause grain development, especially in rapid endosperm nuclear divisions, and can help delay senescence by stimulating sucrose production, thereby maintaining stay-green phenotypes in the late stage of development. In addition, cytokinin can manipulate amino acid metabolism by regulating the distribution of nutrients within the plant and enhancing transport and mobilization [6].

A common wheat breed (*Triticum aestivum*) is an allohexaploid species with three genomes, derived from ancestral species (AABBDD: A genome from Triticum Urartu, B genome from Aegilops speltoides, and D genome from Aegilops tauschii). This genome reflects the complex genetic instructions of wheat, which contains six sets of chromosomes, making it a hexaploid [7]. The complexity of the wheat genome leads to genetic diversity and adaptability, enabling it to grow and survive in a variety of environmental conditions. For example, wheat under drought stress has been classified based on its ability to manage water, reduce transpiration, or improve root depth [6]. Cytokines influence stomatal closure to minimize water loss and can also promote the development and elongation of root growth, allowing for deeper access to water in the soil. With limited water, wheat genotypes that are treated with cytokinins are significantly affected by a reduction in traits such as photosynthesis rate (μmols CO_2_/m^2^/s) by 14% and 10%, compared to 24% and 12% without water deficit. The mean of the membrane stability index for genotypes with cytokine increased by 5% compared to water-deficit genotypes without cytokinins [1,2,3,4,5,6,7,8]. The metabolism of cytokinin in wheat regulates active cytokinin level through dephoshorylation and deribosylation during development processes. These processes include shoot apical meristem, flower, and vascular developments; retaining sink activity to inhibit nitrogen remobilization; and maintaining protein synthesis [9].

Cytokinins play a dual role in plant physiology, regulating both growth and stress adaptation. In fact, cytokinins promote cell division, delay senescence, and modulate nutrient allocation under optimal conditions. However, during drought stress, endogenous cytokinins levels typically decline due to upregulated cytokinin dehydrogenase (CKX) activity, which breaks down cytokinins [3,4,5,6,7,8]. Several observations showed that the application of a foliar spray of cytokinins enhanced the drought tolerance of wheat [9]. The study by [9] concluded that the application of cytokinins in terms of foliar spray enhance drought tolerance by delaying drought caused by the synthesis of cytokinin [9].

The cytokinin expression of the studied wheat through amine acid is crucial for its development. The aim here was to explain wheat genomes (mRNA and proteins) derived from two different databases in terms of their properties and function through the cytokinin riboside 5′-monophosphate phosphoribohydrolase sequences using relative synonymous codon usage (RSCU), molecular weight (g/mol), theoretical isoelectric point, instability index, aliphatic index, and hydrophobicity. The future objective of these results includes beneficial nutrition and industrial applications, such as enhancing digestibility and developing nutritional supplements that can aid in breeding programs [10].

## 2. Results

### 2.1. The Number of Chromosomes for Cytokinin Riboside 5′-Monophosphate Phosphoribohydrolase Genes Through NCBI and Ensembl Plants Databases

**Figure 1:**
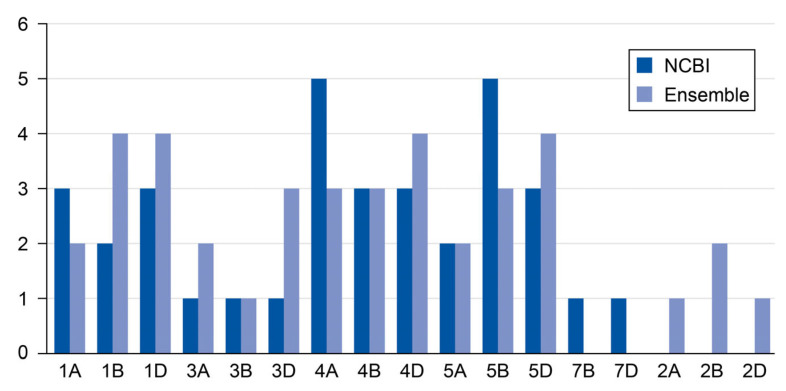
The number of gene sequences in each chromosome across the NCBI and Ensembl databases.

The NCBI database contained 35 genes distributed across different chromosomes, while the Ensembl database contained 38 genes, and both databases indicated the locations of these genes. Figure 1 shows that A, B, and D genomes have different numbers of chromosomes. There were no 2A, 2B, and 2D genomes in the NCBI databases, whereas the Ensembl database contained one gene in both genomes 2A and 2D and two genes in the 2B genome. Some genomes had more genes than other genes. For example, genomes 4A and 5B had five genes from the NCBI database, while genomes 1B, 1D, 4D, and 5D had four genes in the Ensembl database. These gene differences originated from key factors such as the accumulation of genetic differences over time, repeated sequences due to transposable elements (TEs) that could be placed in different locations, gene losses of approximately 10 to 16 thousand genes (as shown in several reports), and the hybridization of wheat from breeding programs [11].

In addition, the cytokinin, riboside 5′-monophosphate phosphoribohydrolase, has multiple members that reflect the functional redundancy for cytokinin metabolism roles across developmental stages. The gene expression of cytokinin riboside 5′-monophosphate phosphoribohydrolase has different cis-regulatory elements. The discrepancy in gene counts between the NCBI and Ensembl databases arises from differences in split or merged genes and annotated genes. For example, NCBI predicted two genes (gene-f and gene-g) whereas Ensembl only annotated one, leading to count discrepancies. In addition, NCBI is preferred for well-characterized genes, while Ensembl excels in exploratory research with novel/predicted genes [3,4,5,6,7,8,9].

### 2.2. The Relative Synonymous Codon Usage (RSCU)

**Figure 2:**
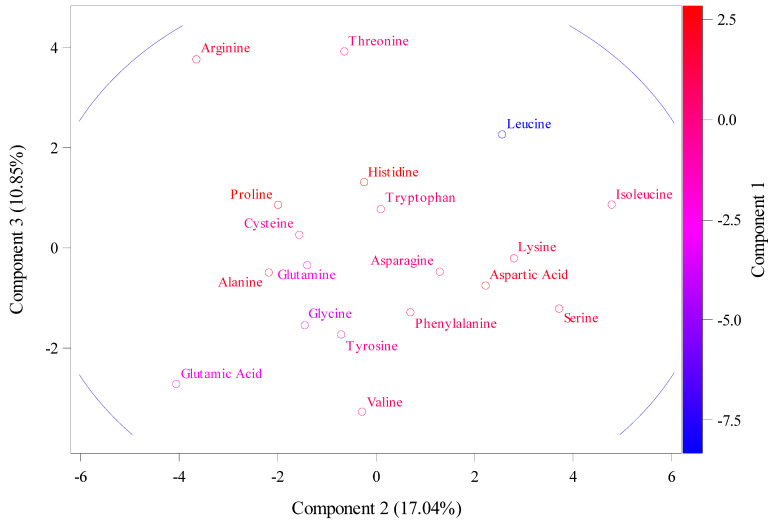
The principal component analysis (PCA) for the relative synonymous codon usage (RSCU) for 35 gene sequences retrieved from NCBI.

The results of amino acids that had RSCU values above 1.6, reflecting a significant over-representation in the following locations: sphenylalanine (TTC), located in 5B chromosomes; Leucine (TTA), located in 5A and 1D chromosomes; leucine (TTG), located in 5B chromosome; leucine (CTC), located in 5D, 7D, and 7B chromosomes; and Leucine (CTA), located in 5D. In addition, other amino acids were Serine (TCT), located in 5D, 1B, and 4A; Proline (CCT), located in 4B; and Threonine (ACG), located in 4D, 5B, 5A, and 4B. However, amino acids that had RSCU values below 0.6 suggesting underrepresentation were leucine (CTA), located in 3A, 5D, 1B, 5B, and 7B; Valine (GTT) located in 4D, 5B, 4A, and 5B; and Proline (CCC) located in 5B, 5D, 5A, 4B, 1B, and 4A. In addition, other amino acids were Threonine (ACC), located in 3A, 4D, 5B, 5A, and 4B; Glutamic Acid (GAA), located in 5B, 5D, 5A, 4A, 1D, 5B, and 7B; Arginine (CGT), located in 3A, 5B, 1B, 5B, and 7D; Serine (AGT), located in 4D, 5B, 5A, 4D, 4B, and 7B; Glycine (GGT), located in 3A, 5B, 5D, 7D, and 7B. Figure 2 shows data from the principal component analysis that illustrate these amino acid patterns. The amino acids that contribute to the main variation are assigned to PC1, with a total variation of about 72.11%. These amino acids include Isoleucine, Leucine, Lysine, Aspartic Acid, Serine, etc., as shown in Figure 2. PC2 and PC3 had total variations of 17.04% and 10.85, respectively, and they were assigned with arginine, glutamic acid, and threonine for PC2, as well as aspartic acid.

### 2.3. The Values of Molecular Weight and Theoretical Isoelectric Point

**Figure 3:**
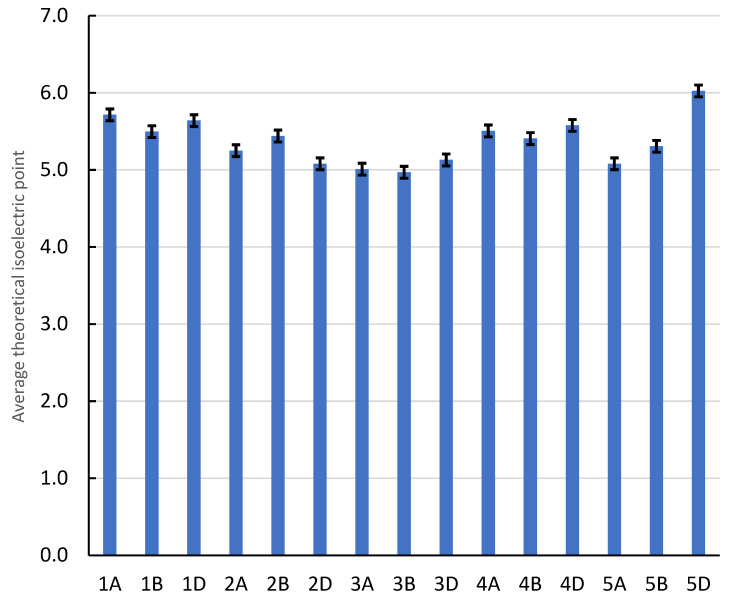
The Y-axis shows average theoretical isoelectric point (pI) for wheat genomes with standard deviation.

**Figure 4:**
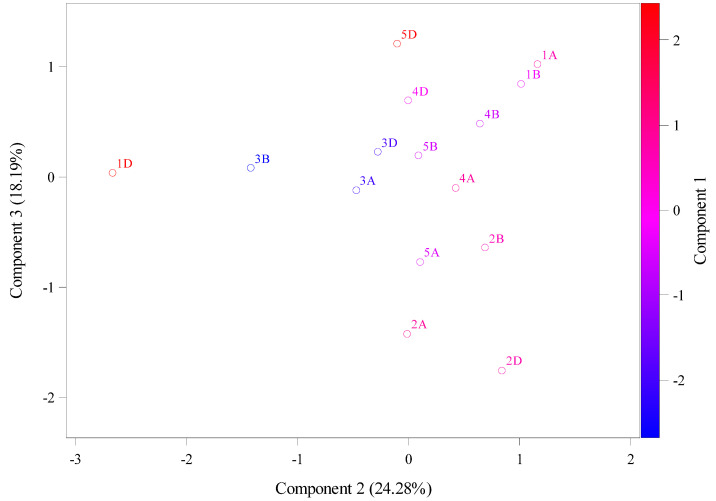
The principal component analysis (PCA) of molecular weight (g/mol), theoretical isoelectric point, instability index, aliphatic index, and hydrophobicity values using the Ensembl database.

Table 1 shows the result values of molecular weight (g/mol), theoretical isoelectric point, instability index, aliphatic index, and hydrophobicity. Theoretical isoelectric point (PI) is the pH that ranged from 4.81 to 6.6 in chromosomes 3A and 4D, respectively (average 5.37 ± 0.297 Figure 3). However, the instability index showed that these two chromosomes (3A and 4D) were at 34.3 and 38.16, respectively. In addition, these two chromosomes (3A, 4D) had aliphatic index values of 88.69 and 89.59, indicating an increase in their stability, and their hydrophobicity values were lower, at 0.04 and −0.04, respectively. This high instability was found in 1D and 5D, with values of 54.16 and 50.36, respectively, indicating a decrease in their stability as shown by aliphatic index values of 92.98 and 88.91. Figure 4 shows data from the principal component analysis (PCA) for amino acid patterns. From this figure, amino acids that explained the main variation are shown to be assigned to first principal component (PC1) analysis, with a total variation of about 58.88%, and these chromosomes are 5D, 4D, 1A, 4B, 3D, etc. The second principal component (PC2) and third principal component (PC3) analyses had total variations of 27.52% and 13.6%, respectively, assigned to 1D, 3B, 3A, 2D, 2A, and 2B.

## 3. Discussion

Cytokinins are important phytohormones encoded by the *LOG* family of genes in wheat and other crops. They play roles in several stages of plant development, including regulating growth and affecting the yield of wheat [12]. It has been reported that the pattern of expression of these genes reflects their roles in growth and reproductive development. In Arabidopsis, this cytokinin acts redundantly at a specific level in the vascular tissues of developing flowers and leaves [13,14].

The importance of relative synonymous codon usage (RSCU) in plant breeding lies in helping breeders understand the mechanisms and functional aspects of wheat genomes; therefore, wheat genomes can be selected for environmental adaptations. The selection of an optimal codon facilitates the transcription of mRNA by avoiding premature termination.

Study [15] showed that the codon usage patterns were different among wheat species because of long-term evolutionary processes, different environment conditions, and mutational processes. In addition, codon bias uses balance to help to distinguish between selection, drift, and mutation for optimal translational efficiency [15].

RSCU values above 1.6 indicate a strong positive codon preference for several codons including TTC, TTA, TTG, CTC, CTA, ATT, and GTC; all of which these are associated with the T-ending codon group. In addition, codons TCT, CCT, and ACG have a C codon, while codons CGC and GGC have a G codon. Thus, these codons can be used for optimizing gene expressions in order to modify some endogenous genes to increase protein production [16]. The importance of principal component analysis here is to identify important amino acids for the cytokinin, riboside 5′-monophosphate phosphoribohydrolase, to make the best decision regarding selection in plant breeding programs.

The isoelectric point of a protein measures the acidity of a solution. The values of theoretical isoelectric point ranged from 4.81 to 6.6 pH. The acidic or basic of proteins is not related to the alkalinity or acidity. The distribution of pI is due to differential composition of amino acids and may be associated with environmental pressure [17]. Research showed that the negative amino acids of durum wheat (Asp and Glu) and positive amino acids (Arg and Lys) in the Glu A1 subunit had a theoretical pI of about 8.66 and lower instability index of about 55.87 [18]. In addition, this study predicted that Glu B3 had the highest value in durum wheat compared to bread wheat [17].

PC1 explains the total variation, highlights the most important amino acids that are associated with the variability of this enzyme. Thus, more attention is paid to these amino acids in the breeding program [19,20]. These results can be applied to wheat research in both nutritional and industrial applications, such as increasing essential amino acids for improve protein quality for human nutrition and enhancing starch from digestible wheat [21].

## 4. Materials and Methods

The DNA and protein sequences of cytokinin riboside 5′-monophosphate phosphori-bohydrolase were retrieved from two different databases, the National Center for Biotechnology Information (NCBI) and Ensembl, as shown in Table 2. All of the studied sequences were filtered and annotated, including genes, regulatory elements, and the location of genes; duplicates, contaminants, and low-quality reads were removed. From the NCBI database, relative synonymous codon usage (RSCU) was individually calculated as the ratio of observed frequency to the expected frequency for each codon, using websites such as https://jamiemcgowan.ie/bioinf/index.html# (accessed on 15 January 2025). Some of codons were excluded from the calculation, such as methionine (ATG), tryptophas (TGG), and stop codon (TAA, TAG, TGA), due to all of them being encoded by one codon and not contributing to the amino acid, thus skewing the results. The formula of calculation RSCU for codon j of amino acid I is calculated using the following formula:

$${RSCU}_{ij=\frac{n_{i}\;x_{ij}}{\sum_{j=1}^{n_{i}}x_{ij}}}\;$$

where *n_i_* is the number of codons that code amino acid _i_, and the *x_i,j_* is the number of occurrences of codon *j*th, and *ij* the degree of codon degeneracy for the *i*th amino acid [22]. The interpretation for *RSCU* values equal to 1 indicated no codon preferences (no bias), while values less than 1 indicated notable negative bias in codon usage. Values above 1.6 reflected significant overrepresentation, while values below 0.6 suggested underrepresentation. Once the RSCU values were determined, all values were further analyzed through SAS 9.4 by principal component analysis.

### 4.1. The Wheat Genome Characterization

Genomes from Ensembl were used to analyze the predicted properties of some of the encoded proteins, such as molecular weight (g/mol), theoretical isoelectric point, instability index, aliphatic index, and hydrophobicity. These characterizations were contacted through web.expasy.org. The theoretical isoelectric point (PI) was the pH value, which can be calculated through the formula pI = (pKa1 + pKa2)/2, where pKa1 refers to the dissociation constant for a group of carboxylic acid, and pKa2 refers to the amino group. Both molecular weight and the isoelectric point help explain biochemical and functional chromosomes of wheat breeding programs. The theoretical isoelectric point helps to determine the stability of protein charge. The instability index refers to the stability of protein. Values less than 40 reflect stable proteins, while those above 40 indicate unstable protein. This instability index can be calculated through the sequence’s length and the weighted sum of dipeptides. The aliphatic index refers to the relative volume, calculated as the based mole percent of amino acid (alanine, valine, isoleucine, and leucine) [23].

### 4.2. Principal Component Analysis

Principal component analysis of the RSCU values and molecular weight (g/mol), theoretical isoelectric point were used through SAS software version 9.4 by using excel file format. In normalization methods, the default methods “PROC PRINCOMP” were used to build correlation matrix that standardizes the variables. The PCA plot can help to analyze values for identify patterns in amino acid or codons that were favored or disfavored in specific or major trends with the reducing complexity of datasets. In general, PCA, as an analytical method, enhances our understanding of protein characteristics, as well as gene expression and evolutionary biology.

## 5. Conclusions

Cytokinin plays crucial roles in wheat breeding, particularly in flowering and yield improvement [24]. This study aimed to analyze the relative synonymous codon usage (RSCU), molecular weight, theoretical isoelectric point, instability index, aliphatic index, and hydrophobicity of wheat cytokinin sequences from two databases. The results showed significant overrepresentation and underrepresentation of certain codons across different chromosomes, which can inform breeding strategies for environmental adaptations. The theoretical isoelectric point and instability index varied across chromosomes, indicating the potential for genetic manipulation. Principal component analysis (PCA) highlighted key amino acids and chromosomes contributing to the variation in RSCU and isoelectric point. These findings underscore the importance of RSCU in understanding wheat genome mechanisms and can aid in developing nutritionally enhanced and industrially beneficial wheat varieties during wheat breeding programs [25].

The importance of calculations for molecular weight, theoretical isoelectric point, instability index, aliphatic index, and hydrophobicity help to characterize substances of polymers and proteins. The molecular weights ranged from 22,282.62 to 30,712.62 g/mol. Thus, molecular weight can provide a meaningful insight into biological function. Other contributing factors that are associated with composition amino acids and values of pI are the rate of nucleotide replacement and mutation [25]. The equilibrium point of a plant tissue in buffers represents the isoelectric point of the proteins. The equilibrium point for potato tissues was about pH 6.4 [17]. The cytokinin and amino acids of wheat need to be further investigated to determine their functional role. Calculating the pI of proteins helps chemists to develop purification programs during protein solubility experiments [25].

## Figures and Tables

**Table 1:** Molecular weight (g/mol), theoretical isoelectric point, instability index, aliphatic index, and hydrophobicity.

Location	Bp	Molecular Weight (g/mol)	Theoretical Isoelectric Point (pI)	Instability Index (II)	Aliphatic Index (AI)	Hydrophobicity (HY)
4A	218	22,991.1	5.38	38.33	93.53	−0.105
2A	243	26,618.74	5.25	40.31	91.44	−0.096
4B	205	22,513.91	5.78	41.69	94.63	0.008
3A	234	25,265.85	5.21	34.3	95	−0.017
1D	215	23,152.61	5.77	54.16	92.98	0.035
3D	241	25,737.38	5.21	33.91	93.49	0.01
1B	268	28,401.58	5.06	38.27	96.12	0.071
1D	239	26,037.71	5.66	43.3	84.1	−0.38
1A	208	22,818.19	6.13	35.3	97.5	−0.045
2B	280	30,712.62	5.71	39.74	95.71	−0.03
2B	246	26,897.94	5.17	38.94	93.09	−0.118
5D	235	24,482.96	6.22	42.68	88.04	0.008
3A	229	24,220.69	4.81	29.84	88.69	0.04
4D	245	25,850.59	6.6	38.16	89.59	−0.049
5D	248	26,288.03	6.23	50.36	88.91	−0.108
5D	247	26,070.71	6.17	41.8	86.52	−0.082
5A	246	26,030.62	5.23	38.94	88.82	−0.063
1B	208	22,819.17	5.98	36.23	97.5	−0.045
5B	241	25,631.11	5.15	35.92	92.24	−0.012
1D	273	28,817.09	5.15	36.73	39.99	0.067
2D	246	26,898.99	5.08	43.64	94.31	−0.085
1A	215	23,217.63	5.3	50	92.05	0.02
5B	241	25,631.11	5.15	35.92	92.24	−0.012
4A	206	22,487.78	5.15	37.95	95.58	0.052
4D	225	23,599.69	5.26	36.46	93.24	−0.106
1B	215	23,221.66	5.45	48	91.12	−0.003
5B	246	26,101.79	5.62	41.79	91.99	−0.002
4D	250	26,176.7	5.28	36.82	87.8	−0.004
3D	226	24,032.55	4.97	28.66	88.98	0.023
5A	245	26,310.95	4.93	39.99	95.51	−0.001
1D	208	22,819.17	5.98	36.23	97.5	−0.045
3B	226	24,032.55	4.97	27.99	88.98	0.023
4A	248	26,111.76	5.99	48.45	88.06	−0.105
5D	249	26,428.14	5.48	43.07	90.48	−0.032
3D	239	25,637.3	5.21	34.82	94.64	0.014
4B	205	22,282.61	5.29	34.03	97.51	0.107
4D	205	22,315.99	5.17	36.73	96.05	0.08
4B	226	23,572.58	5.15	37.49	91.15	−0.125
Mean	233.947	25,058.883	5.455	39.130	90.923	−0.029
StD	19.385	1982.593	0.439	5.748	9.140	0.084

**Table 2:** The DNA and protein sequences of cytokinin from the National Center for Biotechnology Information (NCBI) and Ensembl databases.

	NCBI		Ensembl	
	Gene Symbol ID	Chro.	Gene Symbol ID	Chro.
1	LOC123045576	1A	TraesCS4A02G277200	4A
2	LOC123045860	1A	TraesCS2A02G380600	2A
3	LOC123061754	3A	TraesCS4B02G250400	4B
4	LOC123065219	1A	TraesCS3A02G211100	3A
5	LOC123070388	3B	TraesCS1D02G367400	1D
6	LOC123078846	3D	TraesCS3D02G213900	3D
7	LOC123080662	1B	TraesCS1B02G471300	1B
8	LOC123082392	4A	TraesCS1D02G003500	1D
9	LOC123083722	4A	TraesCS1A02G156100	1A
10	LOC123087480	4A	TraesCS2B02G397600	2B
11	LOC123087481	4A	TraesCS5D02G568400	5D
12	LOC123088143	4A	TraesCS3A02G251500	3A
13	LOC123093512	4B	TraesCS4A02G317900	4A
14	LOC123094293	4B	TraesCS5D02G568500	5D
15	LOC123095076	4B	TraesCS5B02G561400	5B
16	LOC123097019	4D	TraesCS5A02G347400	5A
17	LOC123098787	4D	TraesCS1B02G173200	1B
18	LOC123099424	4D	TraesCS5B02G348600	5B
19	LOC123107636	5A	TraesCS1D02G444500	1D
21	LOC123107637	5A	TraesCS2D02G376900	2D
22	LOC123112988	5B	TraesCS1A02G362500	1A
23	LOC123112989	5B	TraesCS5B02G561300	5B
24	LOC123114455	5B	TraesCS4A02G413200	4A
25	LOC123116784	5B	TraesCS4D02G033800	4D
26	LOC123117873	5B	TraesCS1B02G379700	1B
27	LOC123121452	1B	TraesCS5B02G348400	5B
28	LOC123123922	5D	TraesCS5B02G348300	5B
29	LOC123125354	5D	TraesCS3D02G251900	3D
30	LOC123126254	5D	TraesCS5A02G347500	5A
31	LOC123157422	7B	TraesCS1D02G154700	1D
32	LOC123160435	1D	TraesCS3B02G281000	3B
33	LOC123161849	1D	TraesCS4A02G318100	4A
34	LOC123165646	7D	TraesCS5D02G353600	5D
35	LOC123181086	1D	TraesCS3B02G241600	3B
36			TraesCS4B02G313800	4B
37			TraesCS4D02G310800	4D
38			TraesCS4B02G035700	4B

## Data Availability

The data presented in this study are available from the corresponding author upon reasonable request.
